# Acute myeloid leukemia presenting with hepatic dysfunction: Should induction be dose reduced?

**DOI:** 10.1002/jha2.979

**Published:** 2024-08-07

**Authors:** Satish Maharaj, Simone Chang

**Affiliations:** ^1^ Hematology/Oncology, Department of Oncologic Sciences University of South Florida Tampa Florida USA

**Keywords:** anthracycline, hepatic, leukemia, myeloid, pharmacology

## Abstract

The combination of de novo hepatic dysfunction and AML in a medically fit patient presents an unusual predicament. Cases present with obstructive jaundice and imaging typically shows diffuse hepatosplenomegaly, with some cases visualizing myeloid sarcomas causing biliary ductal dilatation. Guidelines for use of anthracyclines in hepatic dysfunction recommend dose reduction based on bilirubin blood levels, either to 50% or even omitting anthracycline. Randomized data however has shown that reduction of anthracycline in AML induction decreases overall survival and lowers remission rate. This case suggests, along withthe literature reviewed, that some medically fit patients with hepatic dysfunction benefit from and tolerate intensive induction therapy well without toxicity.

1

Acute myeloid leukemia (AML) is the most common acute leukemia in adults. It represents a heterogeneous group of aggressive leukemias; hepatic involvement is less common but well‐described across all ages [1, 2, 3, 4]. Cases present with obstructive jaundice and imaging typically show diffuse hepatosplenomegaly, with some cases visualizing myeloid sarcomas causing biliary ductal dilatation [5, 6]. The combination of de novo hepatic dysfunction and AML in a medically fit patient presents an unusual predicament.

A 36‐year‐old female presented with pancytopenia, jaundice, and a left facial mass. She had no past medical history; past surgical history noted cholecystectomy. Liver function testing showed an obstructive pattern with hyperbilirubinemia 100.9 umol/L (5.9 mg/dL) and mild transaminitis (< 1x upper limit normal); laboratory testing also showed hypofibrinogenemia at 60 mg/dL. Prothrombin time was prolonged at 31.4s (11.8–14.8) with an international normalized ratio of 3.1; activated partial thromboplastin time was within the normal range. Viral serologies for causes of hepatitis were non‐reactive. The initial ferritin level was slightly elevated at 250 ng/mL, but there were normal triglyceride levels and no hyponatremia.

Facial imaging confirmed multiple soft tissue masses in the left head and neck (Figure 1A,B). Abdominal imaging showed hepatomegaly measuring 23 cm and splenomegaly measuring 17 cm, but no discrete masses or biliary obstruction (Figure 1C). The coagulopathy was managed with daily monitoring and transfusion of blood products. Bone marrow biopsy revealed acute myeloid leukemia, with monocytic differentiation and 75% infiltration. The bone marrow was examined carefully without any signs of hemophagocytosis, and overall there was a low probability of hemophagocytic syndrome using the HScore screening test [7].

**FIGURE 1:**
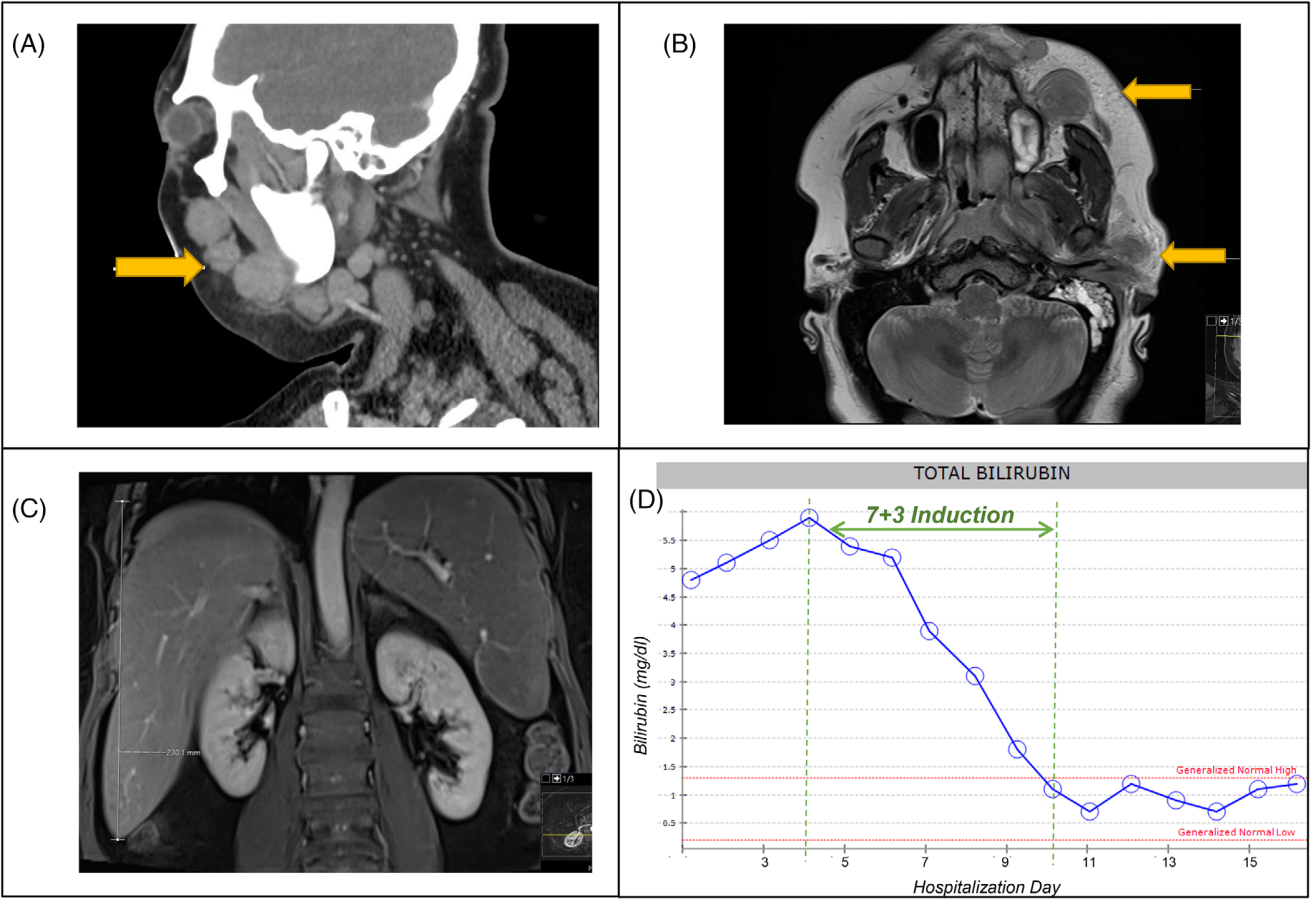
(A, B) Computed tomography and magnetic resonance (MR) imaging confirmed multiple soft tissue masses in the left face and neck, including a 2.7 cm mass anterior to the maxilla, 3.1 cm left parotid mass, and 3.3 cm mass along the lateral margin of the mandible. (C) Abdominal MR imaging showed hepatomegaly measuring 23 cm and splenomegaly measuring 17 cm (craniocaudal dimensions) but no discrete masses or biliary obstruction. (D) Trend in total bilirubin showing response to induction, with a steady decrease continuing until normalization by day 7.

A biopsy of the facial mass also showed malignant monocytic tissue infiltration, consistent with extramedullary myeloid sarcoma.

Phenotypically, the atypical myeloid cells expressed CD34, CD68, CD4, and weakly focal CD56. They were negative for MPO, CD20, CD2, CD3, CD30, ALK1, CD1a, and CD8. A Ki‐67 stain showed a proliferation index of 50%. Their morphology and immunohistologic profile supported a diagnosis of extramedullary acute myeloid leukemia with monocytic differentiation involving the bone marrow as well as soft tissue infiltration (myeloid sarcoma). Next‐generation sequencing detected a pathogenic *NRAS G12V* mutation (Quest Diagnostics LeukoVantage myeloid panel). Cytogenetic testing showed a complex karyotype with 47, XX, t(10;11)(p13;q13.3), der(20)t(1;20)(q21;q13.3), +mar [7]/46, XX [13].

Given the progressing hepatic dysfunction, facial mass, and coagulopathy from AML, in a young and fit patient, immediate therapy was indicated. We discussed the benefits, risks, and alternatives of standard induction therapy, and the patient opted for full‐dose therapy using Cytarabine 200 mg/m^2^ (days 1–7) and Idarubicin 12 mg/m^2^ IV (days 1–3). The hepatic dysfunction steadily improved, with decreasing bilirubin until normalization by day 7 (Figure 1D) and resolution of transaminitis and hypofibrinogenemia as well. There were no undue toxicities and a repeat bone marrow examination showed remission.

The best approach to induction therapy for AML in a newly diagnosed, medically fit patient with hepatic dysfunction is unknown and poses several challenges. Standard AML induction therapy for medically fit patients is a combination of anthracycline and cytarabine (7+3 therapy). This regimen consists of Cytarabine 100–200 mg/m^2^ for days 1–7, and either Idarubicin 12 mg/m^2^ IV or Daunorubicin 60–90 mg/m^2^ IV, on days 1–3. These combination regimens have been standard AML induction therapy for many years. It has been established that maintaining higher doses of anthracycline is essential for the best outcomes [8]. Specifically, randomized studies have shown that induction incorporating higher‐dose anthracycline (Daunorubicin 90 mg/m^2^), compared with 50% lower dosing (Daunorubicin 45 mg/m^2^), leads to significantly higher median overall survival and more complete remissions [9].

Guidelines for the use of anthracyclines in hepatic dysfunction recommend dose reduction based on bilirubin blood levels [manufacturer's labeling, 10]. These recommendations are based on pharmacokinetic models suggesting significant hepatic dysfunction may result in toxicity. The anthracyclines are primarily eliminated by renal and hepatic Aldo‐keto reductase and biliary excretion [10]. For Daunorubicin, a 25% dose reduction is recommended for bilirubin 1.2–3 mg/dL, a 50% dose reduction at 3–5 mg/dL, and avoidance at > 5 mg/dL. For Idarubicin, a 50% dose reduction is recommended for bilirubin 2.6–5 mg/dL, and avoidance at > 5 mg/dL. Cytarabine can also be adjusted to 50% of the total dose for any elevation of aspartate aminotransferase and alanine aminotransferase, or total bilirubin > 2 mg/dL.

We agree the suggested dose reductions seem prudent in patients with chronic comorbid hepatic dysfunction. However, in a young, fit patient with no history of liver disease, hepatic dysfunction on presentation of AML is likely due to leukemic infiltration. Are attenuated doses of induction therapy the best treatment for these cases? Reviewing the literature, there is no consistent approach reported. Indeed, there is a wide range of dosing, from omitting anthracycline therapy to 50% dose reduction to full dosing (Table 1). Alternatively, some cases have attempted to improve hepatic function first, delaying induction using hydroxyurea interim for cytoreduction or employing procedural biliary drains [5], albeit with reports of post‐procedural hemorrhage. Another approach can be replacing anthracycline in initial induction with etoposide, thus allowing improvement in hepatic function and subsequently incorporating dose‐escalating anthracycline in subsequent cycles [2].

**TABLE 1:** Review of selected adult and pediatric cases of acute myeloid leukemia presenting with hepatic dysfunction.

Dosing of anthracycline in induction	Peak total bilirubin	Pathologic features	Other induction therapy administered	Post‐induction therapy	Outcomes reported
Omitted [5, 6, 15, 16]	4.8–41 mg/dL	One case was accompanied by myeloid sarcoma of the thigh; one case with ascites showing AML infiltrate	Dose‐reduced or full dose cytarabine, one case decitabine	Consolidation cytarabine with or without anthracycline, allogeneic HSCT	Death, complete remission/alive at times ranging from post‐consolidation to 2 years post‐transplant
50% dose reduction [2, 3, 4, 11, 12]	4.6–17.9 mg/dL	Four cases with inv [16] (p13q22) / *CBFB‐MYH11* fusion, one case *NRAS* mutation cb	Dose‐reduced or full dose cytarabine, one case dose‐reduced etoposide	Consolidation cytarabine with or without anthracycline, allogeneic HSCT	Complete remission/alive at times ranging from post‐induction to 4 years post‐transplant
Full dose [1, 17, Current case]	5.9–10 mg/dL	One case *NRAS* mutation	Full dose cytarabine	Consolidation cytarabine	Complete remission/alive at times ranging post‐consolidation to 1 year; one case with hepatic recurrence and death at 2 months post‐induction

A survey of British oncologists 25 years ago asked for hypothetical anthracycline dosing in cases of hepatic dysfunction. Similar to the decision‐making reported in Table 1, there was wide variation in the oncologists’ opinions, with some avoiding an anthracycline altogether and others giving full‐dose treatment [13]. The response our patient had to standard induction with no undue toxicity and indeed, reversal of hepatic dysfunction, highlights the need for discussion and data to standardize induction in such cases. Several caveats must be noted to facilitate prompt induction, as bilirubin levels will continue to escalate, potentially making treatment higher risk with delays.

First, a need for rapid diagnostic evaluation when AML hepatic infiltration is suspected. Imaging for compressive etiologies and serology for viral hepatitis should be tested. Hemophagocytic syndrome can be encountered in up to 10% of AML cases and should be part of the work‐up. Key diagnostic features of this include very high ferritin levels (> 5000 ug/L), fever, and bone marrow hemophagocytosis [14].

A hepatic biopsy is not always possible and carries the risk of intrahepatic hematomas and intra‐peritoneal hemorrhaging [Tu]. In cases where it has been possible, liver biopsy showed sinusoidal and portal tract infiltration by blasts accompanied by neutrophils, eosinophils, and monocytes. Additional features reported were cholestasis, macrovascular steatosis, and perisinusoidal fibrosis [3, 4]. In one case, flow cytometry of ascitic fluid showed AML involvement [16].

The small number of cases limits data extrapolation, but some associations were noted (Table 1). First, the presence of coexisting myeloid sarcomas, such as of the soft tissues. Secondly, inv(16) or t(16;16); CBFB::MYH11; AML with abnormalities of chromosome 16 appears overrepresented. Finally, this case and another report *NRAS* activating mutations. Larger analyses are needed to investigate whether certain pathologic features correlate with hepatic involvement from AML.

Secondly, the associated coagulopathy due to the interplay of pancytopenia, hypofibrinogenemia, and liver dysfunction, should be managed closely to mitigate the risk of hemorrhage (especially if procedures are planned). Thirdly, we recognize from the literature that the majority of these cases will show a progressive daily decline in bilirubin levels starting as soon as day 2 of induction. This complicates pharmacokinetic modeling by presenting a dynamic state of clearance instead of a more fixed model (such as hepatic cirrhosis). We suggest therefore that recommendations on dose reductions should be validated not just by pharmacokinetic modeling but also by clinical studies. It would be helpful for high‐volume centers to share their approach and formulate an expert consensus.

In conclusion, dose reductions in AML induction have the potential to decrease overall survival and remission rates. There is a suggestion from our case and others that some medically fit patients with hepatic dysfunction can benefit from and tolerate intensive induction therapy without toxicity. These cases are challenging and the optimal approach requires further research. We acknowledge patients with organ dysfunction are difficult to study in randomized trials but look forward to more data and discussion in attempts to standardize therapy and improve outcomes for these patients.

## CONFLICT OF INTEREST STATEMENT

Dr Maharaj and Dr Chang both have no financial or other relationships that might lead to a conflict of interest.

## FUNDING INFORMATION

The authors received no specific funding for this work.

## ETHICS STATEMENT

The authors have confirmed ethical approval statement is not needed for this submission.

## PATIENT CONSENT STATEMENT

The authors have confirmed patient consent statement is not needed for this submission.

## CLINICAL TRIAL REGISTRATION

The authors have confirmed clinical trial registration is not needed for this submission.

## Data Availability

The authors confirm that the data supporting the findings of this study are available within the article and its supplementary materials.

## References

[1] Mathews E , Laurie T , O'Riordan K , Nabhan C . Liver involvement with acute myeloid leukemia. Case Rep Gastroenterol. 2008;2(1):121–124. 10.1159/000120756 21490850 PMC3075178

[2] Shash HA , Khairy AM . Successful treatment of pediatric acute myeloid leukemia presenting with hyperbilirubinemia secondary to myeloid sarcoma: a case report. Children. 2022;9(11):1699. 10.3390/children9111699 36360428 PMC9688313

[3] Wandroo FA , Murray J , Mutimer D , Hubscher S . Acute myeloid leukaemia presenting as cholestatic hepatitis. J Clin Pathol. 2004;57(5):544–545. 10.1136/jcp.2003.013565 15113866 PMC1770297

[4] Spinelli I , De Santis A , Cesini L , Riminucci M , Corsi A , Forlino M , et al. Acute hepatitis‐like presentation with cholestasis of CBFB‐MYH11‐positive acute myeloid leukemia in an adult male: a case report. J Med Case Rep. 2022;16(1):294. 10.1186/s13256-022-03476-7 35907896 PMC9339180

[5] Lee JY , Lee WS , Jung MK , Jeon SW , Cho CM , Tak WY , et al. Acute myeloid leukemia presenting as obstructive jaundice caused by granulocytic sarcoma. Gut Liver. 2007;1(2):182–185. 10.5009/gnl.2007.1.2.182 20485638 PMC2871626

[6] Jeji PS , Patnaik SK , Behera MK , Narayan J , Sahu MK , Mishra D , et al. Obstructive Jaundice with skin involvement—An unusual presentation of Myeloid Sarcoma. Indian J Pathol Microbiol. 2023;66(4):862–864. 10.4103/ijpm.ijpm_1108_21 38084550

[7] Fardet L , Galicier L , Lambotte O , Marzac C , Aumont C , Chahwan D , et al. Development and validation of the HScore, a score for the diagnosis of reactive hemophagocytic syndrome. Arthritis Rheumatol. 2014;66(9):2613–2620. 10.1002/art.38690 24782338

[8] Teuffel O , Leibundgut K , Lehrnbecher T , Alonzo TA , Beyene J , Sung L . Anthracyclines during induction therapy in acute myeloid leukaemia: a systematic review and meta‐analysis. Br J Haematol. 2013;161(2):192–203. 10.1111/bjh.12233 23398482

[9] Lee JH , Joo YD , Kim H , Bae SH , Kim MK , Zang DY , et al. A randomized trial comparing standard versus high‐dose daunorubicin induction in patients with acute myeloid leukemia. Blood. 2011;118(14):3832–3841. 10.1182/blood-2011-06-361410 21828126

[10] Ofran Y , Tallman MS , Rowe JM . How I treat acute myeloid leukemia presenting with preexisting comorbidities. Blood. 2016;128(4):488–496. 10.1182/blood-2016-01-635060 27235136 PMC5524532

[11] Beck A , Hunter H , Jackson S , Sheridan D . Acute myeloid leukaemia: an unusual cause of biliary strictures. BMJ Case Rep. 2019;12(3):e227821. 10.1136/bcr-2018-227821 PMC6424271 30872340

[12] Tu S , Li M , Fan H , Shi Z , Li X , Song K . Obstruction of the biliary tract as a rare presentation of acute myeloid leukemia: A case report. Oncol Lett. 2023;26(1):300. 10.3892/ol.2023.13886 37323816 PMC10265371

[13] Dobbs NA , Twelves CJ . Anthracycline doses in patients with liver dysfunction: do UK oncologists follow current recommendations? Br J Cancer. 1998;77(7):1145–1148. 10.1038/bjc.1998.190 9569053 PMC2150122

[14] Delavigne K , Bérard E , Bertoli S , Corre J , Duchayne E , Demur C , et al. Hemophagocytic syndrome in patients with acute myeloid leukemia undergoing intensive chemotherapy. Haematologica. 2014;99(3):474–480. 10.3324/haematol.2013.097394 24142998 PMC3943310

[15] Barker JA , Marini BL , Bixby D , Perissinotti AJ . Successful use of high‐dose cytarabine in a patient with acute myeloid leukemia and severe hepatic dysfunction. J Oncol Pharm Pract. 2016;22(6):811–815. 10.1177/1078155215610916 26471735

[16] Rajeswari B , Ninan A , Prasannakumari SN , Parukuttyamma K . Acute myeloid leukemia presenting as obstructive jaundice. Indian Pediatr. 2012;49(5):414–416. 22700670

[17] Goor Y , Goor O , Michalewitcz R , Cabili S . Acute myeloid leukemia presenting as obstructive jaundice. J Clin Gastroenterol. 2002;34(4):485–486. 10.1097/00004836-200204000-00023 11907369

